# Impact of Chronic Multi-Generational Exposure to an Environmentally Relevant Atrazine Concentration on Testicular Development and Function in Mice

**DOI:** 10.3390/cells12040648

**Published:** 2023-02-17

**Authors:** Nicola D. Kolaitis, Bethany J. Finger, D. Jo Merriner, Joseph Nguyen, Brendan J. Houston, Moira K. O’Bryan, Jessica M. Stringer, Nadeen Zerafa, Ngoc Nguyen, Karla J. Hutt, Gerard A. Tarulli, Mark P. Green

**Affiliations:** 1 School of BioSciences, Faculty of Science, University of Melbourne, Melbourne, VIC 3010, Australia; 2 Bio21 Institute, University of Melbourne, Parkville, VIC 3052, Australia; 3 Department of Anatomy and Developmental Biology, Biomedicine Discovery Institute, Monash University, Melbourne, VIC 3800, Australia

**Keywords:** atrazine, testis, multi-generational, endocrine disruptor

## Abstract

A common herbicide, atrazine, is associated with poor health. Atrazine acts as an endocrine disruptor at supra-environmental levels. Little research, however, has been conducted regarding chronic exposure to environmental atrazine concentrations across generations. This study utilized comprehensive endpoint measures to investigate the effects of chronic exposure to a conservative atrazine concentration (0.02 ng/mL), measured in Australian waterways, on male mice fertility across two generations. Mice were exposed through the maternal line, from the pre-conception period and through the F1 and F2 generations until three or six months of age. Atrazine did not impact sperm function, testicular morphology nor germ cell parameters but did alter the expression of steroidogenic genes in the F1, down-regulating the expression of *Cyp17a1* (Cytochrome P450 family 17, subfamily A member 1; *p* = 0.0008) and *Ddx4* (DEAD-box helicase 4; *p* = 0.007), and up-regulating the expression of *Star* (Steroidogenic acute regulatory protein; *p* = 0.017). In the F2, atrazine induced up-regulation in the expression of *Star* (*p* = 0.016). The current study demonstrates that chronic exposure to an environmentally relevant atrazine concentration perturbs testicular steroid-associated gene expression that varies across generations. Future studies through the paternal and combined parental lineages should be undertaken to further elucidate the multigenerational effects of atrazine on male fertility.

## 1. Introduction

Endocrine disrupting chemicals (EDCs) are harmful agents that interfere with hormone signalling pathways, leading to adverse health effects [1]. For decades, EDCs have been widely used without clear understanding of their potential consequences to human health [1]. One EDC of concern is the herbicide atrazine, which is extensively used in Australia [2] and the United States [3]. The effect of atrazine, and other EDCs, is underestimated due to inadequate toxicology testing to determine “safe” levels of exposure [4]. Such testing is not able to reveal the full impact nor more subtle effects of EDCs on the endocrine system, as methodologies do not simulate human environmental exposure. They also fail to consider the impact of transgenerational inheritance [4]. Determining relevant EDC risks would provide a more comprehensive assessment of EDC impact, which can be used to improve legislation and thereby reduce potential health risks [1].

Herbicides are an important class of EDCs to study as substantial quantities are known to contaminate food and waterways [2]. Atrazine (2-chloro-4-ethylamino-6-isopropylamino-1,3,5-triazine) is a common herbicide used in agriculture for corn, maize, sugar cane, and sorghum, as well as on lawns in residential areas to combat weed regrowth [2,5,6]. In 2004, the European Union banned atrazine due to its persistent high levels in groundwater, and potential endocrine health risks (e.g., reproductive disorders) [7]. Despite concern from the European Union, ground and surface waters in the United States of America [3] and Australia [2] remain heavily contaminated with atrazine. This is due to its widespread use and long half-life in surface water and soil, with almost 30% of the original atrazine remaining in the soil after three years [2].

Human exposure to atrazine is most likely to occur through drinking water, with atrazine concentrations frequently measured in water and river sediments in Australia up to 1.65 ng/mL in southeast Australia, and up to 7.6 ng/mL in Queensland [8]. Human epidemiology studies have demonstrated that atrazine exposure is associated with decreased sperm concentration and motility in rural/agriculture areas compared to urban areas [9] but have not been able to establish a causal relationship because of logistical reasons. Hence, animal model experiments can be employed to test causality [4].

Collectively, numerous in vivo rodent studies and in vitro studies have identified adverse effects of atrazine on male reproduction in response to supra-environmental atrazine concentrations (>40 μg/L) [10,11,12,13,14,15,16,17]. The mechanism by which atrazine exerts its effects within the endocrine system is not entirely understood. It is thought that supra-environmental atrazine concentrations (>40 μg/L) can alter enzymatic pathways involved in steroidogenesis [18,19], with inconsistent reports of altered steroidogenic genes, including steroidogenic acute regulatory protein (*Star*), cytochrome P450 family 17, subfamily A member 1 *(Cyp17a1*), hydroxysteroid 17-beta dehydrogenase 3 (*Hsd17β3*), cytochrome P450 family 19, subfamily A member 1 (*Cyp19a1*) and steroid 5 alpha-reductase 1 (*Srd5α1*) [12,19,20,21,22,23]. However, the most consistent alterations in steroidogenesis from supra-environmental atrazine exposure (>40 μg/L) is an up-regulation in *Cyp19a1* expression, and a down-regulation in *Srd5α1* expression within the testis [19,21,24,25,26], that can lead to anti-androgenic and pro-oestrogenic effects [18,19]. 

Thus, atrazine is designated as an anti-androgenic EDC, due to directly inhibiting the expression of the *Srd5α1* gene, that encodes the enzyme 5α-reductase type 1 [19]. Atrazine inhibition of 5α-reductase reduces the conversion of testosterone to 5α-dihydrotestosterone (DHT), resulting in a reduction in DHT levels [19]. Atrazine is also known to have an oestrogenic effect via an increase in the expression of the *Cyp19a1* gene, that encodes the enzyme aromatase [19]. Atrazine promotion of aromatase increases the conversation of testosterone to oestrogen, resulting in an increase in oestrogen levels [19]. Atrazine-induced increases in aromatase expression are evident in amphibians [24], mice [19,21,25] and in vitro with human oestrogen sensitive tissues [26]. The mechanism behind this action remains unclear; however, it is suggested that atrazine inhibits the activity of phosphodiesterase [27], which regulates cyclic adenosine monophosphate (cAMP), that itself increases the expression of aromatase [28].

A perturbation in steroidogenesis can adversely influence the physiology of reproductive organs [29,30]. Specifically, alterations in endocrine hormone levels in response to atrazine exposure can delay pubertal onset in males [10], disrupt sperm production/function [10,11,19,20,31] and result in developmental disorders of reproductive organs, including cryptorchidism and hypospadias [11,12,13,14,22,32,33,34]. Such impacts are reported in disparate species, including fish, amphibians, reptiles and mammals [15].

Notably, numerous studies have illustrated reduced sperm number and functionality in response to atrazine exposure [10,11,19,20,31]. However, very few of these studies have looked at how developmental steps of spermatogenesis influence sperm defects. This is essential as alterations in germ cell populations combined with alterations in steroidogenesis may be the cause of the previously reported sperm defects.

In Australia, the National Health and Medical Research Council (NHMRC) have set the “safe” drinking water limit of atrazine at 20 ppb [35]. This limit is based upon results of a 1990s study showing a no-observed-effect level (NOAEL) in adult male rats being exposed to 0.5 mg/kg bw/day of atrazine over a period of two years [36,37]. Caution should be taken when extrapolating these data, as evidence of safety to humans via traditional toxicity studies often fails to provide insight into the full scope of endocrine effects, as these studies overlook periods of reproductive development, such as during fetal development and puberty, which are highly susceptible to the impacts of EDC exposure [38]. Furthermore, these studies have not interrogated the impacts of atrazine exposure across generations. Humans are exposed to EDCs chronically, with individuals potentially exposed from fetal life to conception and gestation of their offspring and beyond [38]. Consequently, multigenerational studies of atrazine need to be undertaken to assess the cumulative effects of in utero, postnatal, and adult exposure on an individual. It is also essential to analyze the possibility of inheritable effects across generations [39], especially as transgenerational changes in the sperm epigenome are known [17].

Lastly, when studying EDCs, it is critical to determine accurate and relevant exposure doses and concentrations [40]. Several studies have utilized supra-environmental doses of atrazine to demonstrate an effect [10,11,16,21,31,32,33,34,41]. Whereas, data regarding environmental levels are scarce, as low levels humans receive from waterways are assumed to be safe [1]. Nevertheless, some EDCs may have non-linear dose response relationships, with unknown endocrine effects below the NOAEL [40], meaning there could be novel findings on atrazine’s impact to steroidogenesis when utilizing biologically relevant doses.

The current study aimed to address the lack of information surrounding chronic exposure to an environmentally relevant atrazine concentration, as well as exposure across multiple generations via the maternal lineage. Mice were chronically exposed to environmentally relevant atrazine (0.02 ng/mL) in drinking water for two generations and impacts on male fertility were measured by assessing reproductive organ morphology, germ cell and sperm characteristics, as well as steroidogenic gene expression.

## 2. Materials and Methods

### 2.1. Animals and Experimental Design

Experimental procedures were approved by the Monash University Animal Ethics committee (Ethics approval number: 23736) and performed in accordance with the National Health and Medical Research Council’s Australian Code of Practice for the Care and Use of Animals in Science [42]. All mice were housed in the Monash University Animal Research Laboratories in a temperature-controlled environment with 12 h light and 12 h dark cycles. Mice were fed ad libitum a soy-free diet (SF06-053, Specialty Feeds, Perth, WA, Australia) to remove any endocrine effect of dietary phytoestrogens.

The atrazine concentration of 0.02 ng/mL was chosen as it represents the conservative environmentally relevant concentration of atrazine in Australian waterways [8]. Atrazine (CAS number: 1912-24-9; 100% purity; Sigma-Aldrich, St. Louis, MO, USA) powder was dissolved in DMSO (Sigma-Aldrich) and then diluted to a 0.02 ng/mL concentration in autoclaved Milli-Q water. Both the atrazine drinking water (ATZ) and vehicle control drinking water contained the same concentration of DMSO (0.0000001% *v*/*v*).

Female C57BL/6J mice (4 to 6 weeks old; n = 18) were purchased from the Monash University Animal Research Platform (MARP, Melbourne, Australia) and began exposure to ATZ or control drinking water three weeks prior to mating with unexposed males (C57BL/6J/MARP). F0 females continued the ATZ or control treatment post birth, during the lactation phase and up to weaning their pups at three weeks of age. Offspring from the F1 were weaned at three weeks of age (n > 8 litters), and continued exposure to ATZ or the control via their drinking water. F1 males were culled at three and six months for post-mortem tissue collection. To produce the F2, treated F1 females were mated with unrelated unexposed male mice to avoid confounding effects of ATZ on spermatogenesis. Exposure during pregnancy, lactation and weaning continued as described above. F2 offspring continued exposure to three or six months when they were culled for post-mortem tissue collection (n > 8 litters).

### 2.2. Puberty Determination

F1 and F2 males were assessed for pubertal timing. From 20 days of age, F1 and F2 males underwent daily assessment for prepuce separation from the glans penis [43]. The day of prepuce separation, indicating puberty, was recorded.

### 2.3. Post-Mortem Tissue Collection and DEXA Analysis

At either three-months or six-months, F1 and F2 males were killed via inhalation and overdose of isoflurane (Pharmachem, Eagle Farm, QLD, Australia). Males were analyzed across litters (n > 7 L per treatment), with n > 10 males per treatment. Dual-energy X-ray absorptiometry (DEXA) scans were performed on both three and six-month cohorts, in the F1 and F2, as per the manufacturer’s instructions. Briefly, using a Lunar PIXImus machine (LUNAR, Fitchburg, WI, USA) and Lunar PIXImus2 2.10 software, the percentage of fat and muscle, fat and lean mass, bone mineral density (BMD) and bone mineral content (BMC) were obtained. Calibration was performed using a Quality Control Phantom mouse before each run. Body weight was recorded, the epididymides were dissected and incubated under pre-warmed mineral oil (Sigma-Aldrich) prior to sperm collection and analysis (n = 7 to 10 males per treatment per generation). The testes, seminal vesicle, liver and gonadal fat were dissected and weighed. Testes were snap-frozen in liquid nitrogen and stored at −80 °C for gene expression analysis or were fixed in Bouin’s fixative (Sigma-Aldrich) for histological analysis.

### 2.4. Sperm Analysis

Sperm motility was assessed using a Hamilton Thorne computer-aided semen analysis (CASA) machine (MouseTraxx, Chicago, IL, USA), as previously described [44]. Sperm from the cauda epididymides and vas deferens were collected from F1 and F2 six-month old male mice sperm measurements included total motility (%), progressive motility (%) and rapid velocity distribution (%). At least 1000 sperm were measured per mouse.

### 2.5. RNA Isolation and cDNA Synthesis

Snap-frozen testes from the six-month-old mice were processed for gene expression analysis in both the F1 and F2. RNA was extracted using TRIzol reagent (Invitrogen, Mulgrave, VIC, Australia) and phase lock tubes (5Prime) as per the manufacturer’s instructions, and as previously described [45]. Extracted RNA was DNase treated using the TURBO DNA-free kit (Invitrogen) following the manufacturer’s specifications. The total RNA concentration and quality was assessed using the NanoDrop One/One^C^ UV–Vis Spectrophotometer (Thermo Fisher Scientific, Scoresby, VIC, Australia). The DNase-treated RNA was reverse transcribed into complementary DNA (cDNA), using Superscript III First-Strand (Invitrogen) as per the manufacturer’s instructions, and as previously described [19]. Briefly, RNA (800 ng) was annealed to 1 µM primers (oligoDT) at 65 °C for 5 min, followed by a master-mix containing Superscript III enzyme, and further incubated at 50 °C for 50 min, and 85 °C for 5 min.

### 2.6. Quantitative RT-PCR

Quantitative real time PCR (qRT-PCR) was performed in triplicate with 10 µL reaction volumes on 384 well reaction plates using 2X SYBR Green (Applied BioSystems, Scoresby, VIC, Australia) and the Viia^TM^7 thermocycler (Applied BioSystems). Both minus RT and water controls were included on each reaction plate. A QC sample (2 µL from each cDNA sample) with primer *β-actin* was added to each plate to act as a quality control between reaction plates. Primers were synthesized by IDT (Integrated DNA Technologies, Singapore) and sequences are listed in Table A1. The PCR cycle conditions were 50 °C for 5 min, 95 °C for 10 min, then amplified for 40 cycles, where each cycle included denaturation for 15 s at 94 °C, annealing for 30 s at 60 °C and extension for 30 s at 72 °C, followed by a final extension at 72 °C for 5 min. Relative gene expression was calculated using the Pfaffl method with known primer efficiencies [46]. Target genes were normalized to the geometric mean of three housekeepers (Beta actin, *β-actin*; TATA-box binding protein, *Tbp*; Ribosomal Protein L19, *Rpl19*), and the results were represented as a fold change relative to the control.

### 2.7. Histology of Testis Sections

Bouin’s fixed testes from the six-month-old F1 mice were washed twice for 30 min in 1 × phosphate buffered saline (PBS), before being placed in 70% ethanol and stored at 4 °C until processing [47]. Testis samples were then embedded in paraffin wax, sectioned at 5 µm thickness and stained with Harris hematoxylin and eosin Y (H&E) using a standard protocol and as previously described [48]. Sections were visualized using the Zeiss Axioplan 2 microscope, equipped with the Zeiss Axiocam 308 color camera. Images of seminiferous tubules were captured at 100× magnification and analyzed using ZEN 3.4 (blue edition, Zeiss, Macquarie Park, NSW, Australia). For each animal, one section was randomly selected per slide, and the section was systematically sampled from a random start. Every third image at 100× was quantified. Using ZEN 3.4, the epithelial height (µm), tubular diameter (µm^2^) and the percentage of tubule versus interstitial space was measured. Tubules without a clear lumen were excluded from the analysis and area with tubule shrinkage was removed from the final calculation. In total, >250 tubules were analyzed across >5 sections per animal.

### 2.8. Immunohistochemistry of Testis Sections

Bouin’s fixed testis sections used for H&E were used for immunohistochemistry. Tissue dewaxing, rehydration, antigen retrieval and primary and secondary antibody staining protocols were performed using a published protocol [48]. In short, antibody staining was undertaken using four primary antibodies (SOX9, EMD Millipore, #AB5535, rabbit; P-HH3, EMD Millipore, Burlington MA, USA, #AB10543, rat; GATA4, Invitrogen eBioscience, #14-9980-82, rat; DDX4, Abcam, Melbourne, VIC, Australia, #AB13840, rabbit), 10% normal goat serum (Sigma-Aldrich) in 1 X PBS (pH 7.4) followed by one of two secondary antibodies (488 IgG anti-rat, Invitrogen, #SA5-10018; 555 IgG anti-rabbit, Abcam, #AB96892), in 10% normal goat serum in 1 X PBS. This was followed by a counterstain with DAPI and mounting under glass coverslips with fluorescent mounting media (Sigma-Aldrich). The negative controls involved sections with PBS only, secondary antibody only, or an antibody isotype primary antibody control. For visual presentation, images were captured on a Nikon A1R confocal microscope (Nikon, Tokyo, Japan) [49]. For quantification, images were captured via the Zeiss Axioplan 2 microscope, equipped with the Zeis Axiocam 305 mono camera, and analyzed using ImageJ. Measurements involved the number of proliferating cells per total testis section (P-HH3 positive cells), the number of Sertoli cells per unit area (SOX9 positive cells), and the percentage area of *Ddx4* expression per tubule (DDX4 positive cells). For each animal, one section was randomly selected per slide, and the section was systematically sampled. Every third image at 100× magnification was used for quantifying the area of *Ddx4* expression and the number of Sertoli cells. Using ImageJ, the total area of DDX4 staining per tubule was outlined, measured, and represented as the percentage of DDX4 area per tubule. Using ImageJ, the number of Sertoli cells was manually counted, and expressed as the number of cells per unit area. The number of proliferating cells in an entire tissue section was manually quantified using ImageJ and was normalized to the total area.

### 2.9. Statistical Analyses

Data comparisons were tested for normality and homogeneity using boxplots and the Shapiro–Wilk testing. A Mann–Whitney U test was used to analyze age at puberty and compare the differences between the ATZ and control groups, with the statistical significance set at *p* < 0.05. A Fisher’s exact test was used to analyze the sex ratio and compare differences between the ATZ and control groups, with the statistical significance set at *p* < 0.05. Unpaired *t*-tests were used to analyze the remaining in vivo data and compare differences between the ATZ and control group within each generation for all in vivo endpoints, with the statistical significance set at *p* < 0.05. All data endpoints were analyzed using Graph Pad Prism (9.3.1) and were presented as mean ± SEM.

## 3. Results

### 3.1. ATZ Exposure Did Not Modify Litter Characteristics or the Timing of Puberty Initiation

Atrazine exposure had no effect on litter size, litter weight, sex ratio or mortality rates within or across generations (data not shown). Pubertal development is hormonally sensitive; therefore, the time to puberty was assessed in the F1 and F2 cohorts as a functional readout of hormone activity. There was no change in the age of pubertal onset in the F1 and F2 mice treated with atrazine (data not shown, combined average F1 and F2 (control = 25.89 ± 0.51 days; ATZ = 27.8 ± 0.59 days).

### 3.2. ATZ Exposure Did Not Affect Body and Organ Weighs

Atrazine exposure did not cause a significant change in body weight in the 3-month or 6-month cohort in the F1 (*p* > 0.05; Table 1) or F2 generation (*p* > 0.05; Table 2). There were also no differences in testis, seminal vesicle, liver, or gonadal fat weights from atrazine exposure in the 3-month or 6-month cohort in the F1 and F2 generation (*p* > 0.05; Table 1 and Table 2).

### 3.3. ATZ Exposure Did Not Affect DEXA Parameters

Atrazine exposure in the F1 male offspring was not associated with a significant change in bone mineral density (BMD), bone mineral content (BMC) or the percentage of fat and muscle in the 3-month or 6-month cohort (*p* > 0.05; Table 3). Equally, no changes were evident in the F2 for the 3-month and 6-month cohorts (*p* > 0.05; Table 4).

### 3.4. ATZ Exposure Did Not Result in Gross Morphological Changes in the Testis

**Figure 1:**
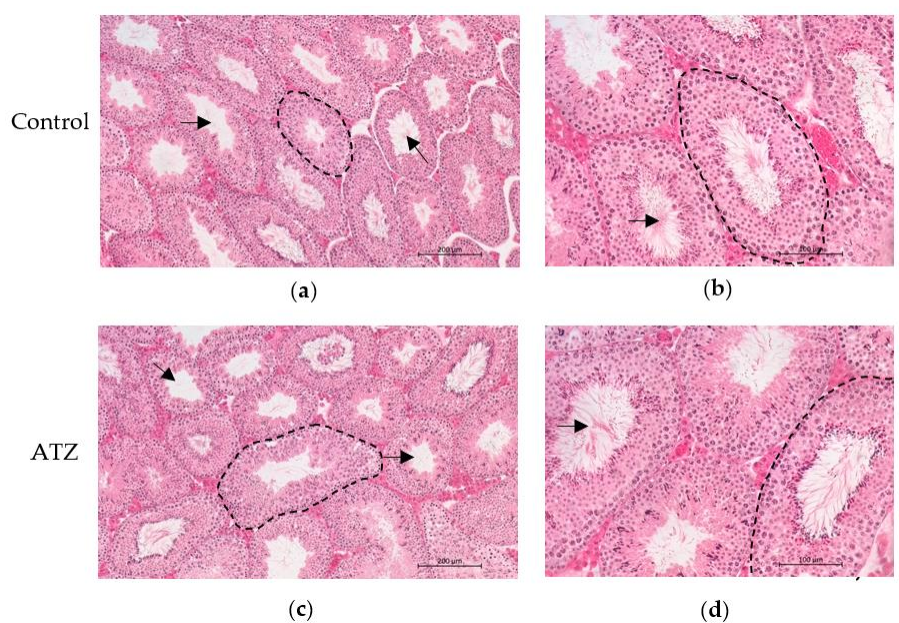
Representative images of H&E-stained testis section from F1 mice exposed to 0.02 ng/mL atrazine or the control for 6 months. (**a**) control (×100), (**b**) control (×200), (**c**) ATZ (×100), (**d**) ATZ (× 200). No visual difference in gross morphology was observed between treatment groups. Scale bar at 100× is 200 µm, and at 200× is 100 µm. Arrow indicates the tubule lumen and the dashed line indicates the basement membrane of the tubule.

To determine if atrazine exposure resulted in any gross morphological changes within the testis of the F1 mice, testis sections from the 6-month cohort were stained with H&E. Qualitative appraisal of testis sections revealed no changes in gross morphology between the control and ATZ groups (Figure 1).

### 3.5. ATZ Exposure Did Not Affect Sperm Characteristics

To characterize the effects of atrazine exposure on sperm parameters, CASA analysis was performed. Atrazine exposure in the F1 and F2 male offspring did not result in a significant change in the percentage of motile sperm (total or progressive) and no change to rapid velocity distribution in the 6-month cohort (*p* > 0.05; Table 5).

### 3.6. ATZ Exposure Altered Early Steroidogenic Gene Expression

**Figure 2:**
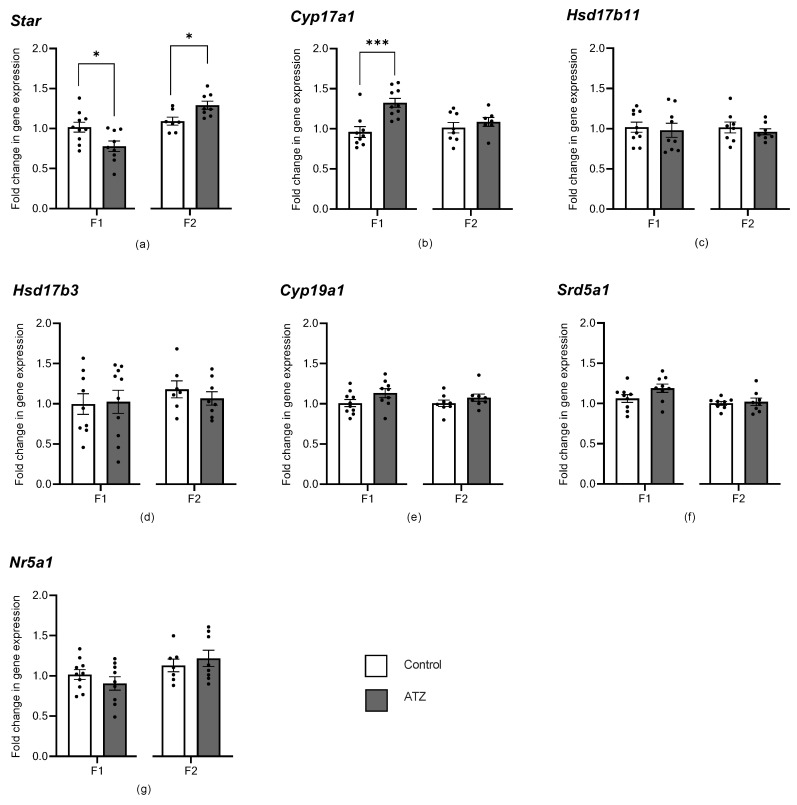
F1 and F2 testis gene expression assessed by qRT-PCR. (**a**) *Star*, Steroidogenic acute regulatory protein, (**b**) *Cyp17a1*, Cytochrome P450 family 17, subfamily A member 1, (**c**) *Hsd17β11*, Hydroxysteroid 17-beta dehydrogenase 11, (**d**) *Hsd17β3*, Hydroxysteroid 17-beta dehydrogenase 3, (**e**) *Cyp19a1*, Cytochrome P450 family 19, subfamily A member 1, (**f**) *Srd5α1*, Steroid 5 alpha-reductase 1 and (**g**) *Nr5a1*, Nuclear receptor subfamily 5 group A member 1 (SF-1). Genes were normalized to the geometric mean of *B-actin*, *TBP*, and *Rlp19*. Results were represented as a fold change in gene expression relative to the control, using the Pfaffl method [46]. Data are means ± SEM. Significance between the control (n = 8–10) and ATZ (n = 8–10) is indicated by an asterisk (* *p* < 0.05 or *** *p* < 0.001). There were >4 L per treatment, per generation.

To characterize the effects of atrazine exposure on testis gene expression, transcript levels of genes related to steroidogenesis were measured in the six-month cohorts via qRT-PCR. In the F1, there was a significant decrease in the expression of *Star* in the atrazine-exposed male testes compared with the control male testes (Figure 2, *p* = 0.017), as well as a significant increase in the expression of *Cyp17a1* (Figure 2, *p* = 0.0008). In the F2, however, there was a significant increase in the expression of *Star* in the atrazine-exposed male testes compared with the control male testes (Figure 2, *p* = 0.016). No significant difference in gene expression was observed for all other genes in the F1 and F2 (*p* > 0.05; Figure 2).

### 3.7. ATZ Exposure Altered Expression of Germ Cell Marker, Ddx4

**Figure 3:**
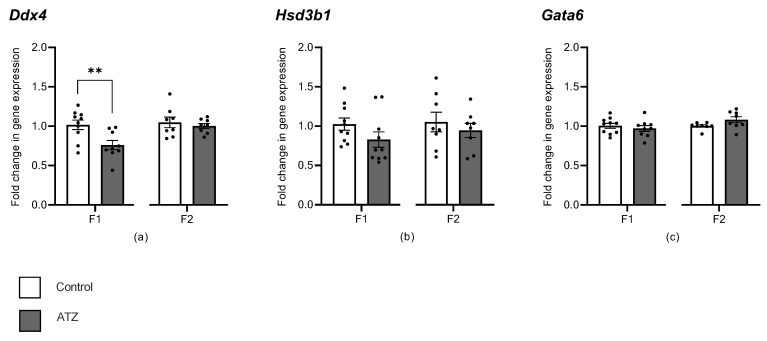
F1 and F2 testis gene expression assessed by qRT-PCR. (**a**) *Ddx4*, DEAD-box helicase 4–germ, cells, (**b**) *Hsd3b1*, Hydroxy-delta-5-steroid dehydrogenase, 3 beta-and steroid delta-isomerase 1–Leydig cells, and (**c**) *Gata6*, GATA binding protein 6–Sertoli cells. Genes were normalized to the geometric mean of *B-actin*, *TBP*, and *Rlp19*. Results were represented as a fold change in gene expression relative to the control, using the Pfaffl method [46]. Data are mean ± SEM. Significance between the control (n = 8–10) and ATZ (n = 8–10) is indicated by an asterisk (** *p* < 0.01). There were >4 litters per treatment, per generation.

To characterize the effects of atrazine exposure on testis gene expression, transcript levels of genes related to cell sub-types within the testis were measured via qRT-PCR. Atrazine exposure in the 6-month cohort of the F1 decreased the expression of the germ cell marker, *Ddx4* (Figure 3, *p* = 0.007). However, in the F2 in the 6-month cohort, there was no change in *Ddx4* expression in the atrazine-exposed male testes (*p* > 0.05; Figure 3). Expression of *Hsd3b1* (Leydig cell marker) and *Gata6* (Sertoli cell marker) was not altered by atrazine exposure compared with the control 6-month-old males in the F1 and F2 cohorts (*p* > 0.05; Figure 3).

### 3.8. ATZ Exposure Did Not Affect Seminiferous Tubule Measurements 

To associate the F1 qRT-PCR decrease in *Ddx4* with biological changes in seminiferous tubule characteristics, testis H&E sections were analyzed. There was no effect of atrazine exposure in the 6-month F1 cohort on the epithelium height, tubule diameter or the percentage of tubule vs. interstitial space (*p* > 0.05; Table 6). 

### 3.9. ATZ Exposure Did Not Affect the Area of Ddx4 Positive Cells, Sertoli Cell Number or the Number of Proliferating Cells

**Figure 4:**
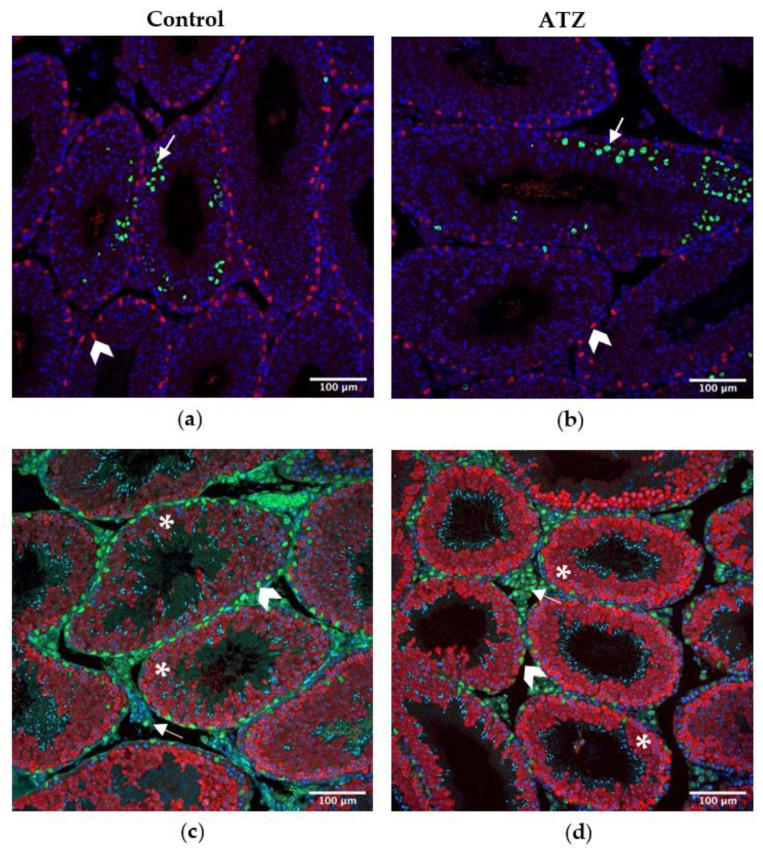
Representative immunofluorescence of testis sections at 200X of the F1 control (**a**,**c**) and ATZ exposed (**b**,**d**) 6-month cohort. (**a**,**b**) immunolabelling of SOX9 (Sertoli cells, red) and of proliferating cells with an antibody raised to phospho-histone H3 (P-HH3, green), with a DAPI counterstain (showing the nuclei, blue). White arrows indicate proliferating cells and white arrow heads indicate Sertoli cells (**c**,**d**) immunolabelling of GATA4 (Sertoli and Leydig cell, green) and of DDX4 (germ cells, red), with a DAPI counterstain (showing the nuclei, blue). White arrows indicate Leydig cells, white arrowhead indicates Sertoli cells and white asterisks indicate germ cells. Scale bar = 100 µm. ATZ = atrazine.

**Figure 5:**
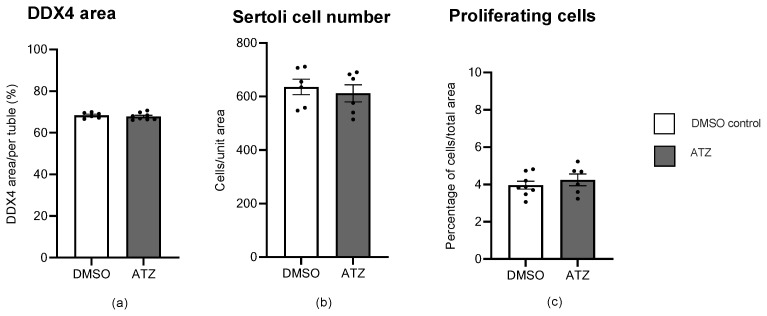
Analysis of atrazine exposure on the number of cell sub-types within the testis at 6 months of age in the F1 cohort. (**a**) DDX4 area was represented as a percentage area per tubule. (**b**) Sertoli cells were normalized to a set area and are represented as number of cells per unit area. (**c**) The number of proliferating cells were normalized to the total testis section area and represented as cells per total area. No significant differences were found between the control (n = 6) and ATZ (n = 6–8) treatment for all parameters. Data are mean ± SEM. There were > 3 L per treatment.

To further investigate the decrease in *Ddx4* transcription in atrazine-treated F1 mice, immunohistochemistry was performed on testis sections with antibodies raised to markers of cell sub-types (Figure 4). Atrazine exposure to the six-month F1 cohort did not cause a significant change in the area of DDX4 positive germ cells, the number of proliferating cells, or Sertoli cells (*p* > 0.05; Figure 5).

## 4. Discussion

This study demonstrates that chronic exposure to an environmentally relevant atrazine concentration across multiple generations does not alter the age at onset of puberty, body or organ weight or sperm motility. However, chronic low level atrazine exposure altered the expression of genes involved in testicular steroidogenesis, *Star* and *Cyp17a1*, and a germ cell marker, *Ddx4*, though changes only persisted into the F2 generation for *Star*. Whilst it was anticipated that exposure to environmentally relevant concentrations would not result in severe impacts on male fertility, it is notable that an atrazine concentration as low as 0.02 ng/mL can change steroidogenic gene expression, and this may result in subtle changes in the endocrine function of the testis. While these results are reassuring, they do not negate the possibility that small, but significant changes in endocrine profiles could change behaviour and thus, ultimately, fertility. 

### 4.1. Atrazine Affects Steroidogenesis

This study is the first to identify that chronic exposure to an environmental atrazine concentration can alter the transcript levels of steroidogenic genes in the mouse testis. In F1 and F2 cohorts, changes were observed in the expression levels of *Star*, a gene responsible for promoting steroidogenesis by allowing the movement of cholesterol into the mitochondria [50]. Unexpectedly however, these changes in *Star* were inconsistent across generations, with a decreased expression in the F1 and an increased expression in the F2. This follows on from previous research that has shown conflicting changes in *Star* expression in response to atrazine. In two similar rodent studies, pregnant rats exposed to a range of atrazine doses (10, 70, and 100 mg/kg) resulted in an up-regulation of *Star* gene expression in male offspring [23], while offspring of pregnant mice exposed to atrazine at 100 mg/kg had down-regulated the expression of *Star* [22]. These data suggest that atrazine can act inconsistently on *Star* expression at supra-environmental and environmentally relevant doses. This highlights the need for further investigation of atrazine impacts on steroidogenesis and whether the fold changes observed manifest as biological changes in the steroid pathway.

Additionally, in the F1 cohort, there was an increase in *Cyp17a1* expression, which is also involved in an initial step in steroidogenesis, converting progestogens to androgens (DHEA and androstenedione) [51]. Previous literature reports conflicting expression levels of *Cyp17a1* in response to atrazine exposure. An in utero study exposing pregnant rats to a supra-environmental atrazine dose (100 mg/kg) demonstrated an increase in *Cyp17a1* expression [23]. Whereas, another rat exposure study utilizing the same atrazine dose and a similar in utero treatment window discovered that contrastingly, atrazine induced a down-regulation of *Cyp17a1* expression [21]. These two studies exemplify the complexity of endocrine control and the inconsistent effects of atrazine effects on steroidogenesis.

Interestingly, neither *Cyp19a1* nor *Srd5a1* genes encoding enzymes previously known to be altered by supra-environmental atrazine exposure in rodent models were altered in the present study [19,21,25]. At supra-environmental levels, atrazine exposure increases *Cyp19a1* gene expression [19], which is responsible for converting testosterone to oestrogen, as well as decreasing the expression of the 5*α*-reductase gene, *Srd5a1* [19], responsible for converting testosterone to dihydrotestosterone. 

Unexpectedly, the present study observed changes in steroidogenesis genes (*Star* and *Cyp17a1*) involved in the initial steps of the pathway, whereas previous studies identified changes in later steps in steroidogenesis (*Cyp19a1* nor *Srd5a1*). The *Star* gene product is primarily involved in the rate-limiting initiating step of steroidogenesis, the transport of cholesterol into the mitochondria [50,52,53], while complete inhibition of *Star* expression reduces steroid synthesis but does not eliminate it [53]. This suggests that in addition to *Star*, there are likely other factors involved in the transport of cholesterol into mitochondria [53]. Such unknown factors could be compensating for the subtle and inconsistent changes in *Star* expression observed in the present study, to ensure end products during later stages of steroidogenesis remain at normal levels. It is important to note, however, that previous studies have not identified similar changes in *Star* expression without evidence of later-stage changes in steroidogenesis. This novel finding could provide insight into a new mechanistic action of how atrazine impacts steroidogenesis, highlighting that different steroidogenic genes may respond to different doses of atrazine. However, while these steroidogenic changes at the transcript level are significant, they are still subtle, and therefore, may not have any functional consequences to steroidogenesis.

### 4.2. Atrazine’s Impact on Testis Cell Sub-Types

The present study demonstrated, for the first time, that environmental atrazine concentrations cause a decrease in the expression of the germ cell marker *Ddx4* in an F1 cohort. However, no change in *Ddx4* expression was observed in F2, neither was there any change in *Gata6* (Sertoli cells) and *Hs3b1* (Leydig cells) expression in F1 and F2. Previous studies have shown that supra-environmental atrazine can alter the germ cell population, with an increased number of abnormal germ cells in Japanese medaka fish [54], and a decrease in *Ddx4* expression in largemouth bass fish [55]; however, there have been no studies performed on rodent models, with similar atrazine concentrations and for similar exposure periods.

The decrease in F1 *Ddx4* may reflect a fundamental change in the proportion of germ cells [56], or represent a subtle defect in germ cell function. However, the germ cell environment was further analyzed via histological measurements (e.g., tubule diameter and epithelial height), as well as specific immunolabelling of DDX4 positive germ cells and SOX9 positive Sertoli cells, as the number of Sertoli cells can dictate the number of germ cells in an epithelium [57]. None of these parameters, including the epithelial height, tubule diameter, the area of DDX4 positive germ cells per tubule and the number of Sertoli cells were changed from environmental atrazine exposure. In contrast, previous studies have shown distinct testis morphological changes to supra-environmental atrazine exposure, including a reduction in seminiferous tubule lumen, increased apoptotic cells and irregular shaped Leydig cells, during a 40-day exposure to 50 mg/kg of atrazine in adult rats [34], as well as seminiferous tubule dilation from a 40-day exposure to 200 mg/kg of atrazine in adult rats [58]. However, the most likely explanation for previous testis morphological changes is from alterations to steroidogenesis caused by supra-environmental levels of atrazine, disrupting the circulating and intra-testicular levels of oestrogens and androgens [34,58].

To further explore functional changes in testis function, this study analyzed the proliferation of germ cells via immunolabelling for phospho-histone H3 (P-HH3), as well as analyzed various sperm measurements via CASA. There was no change observed in the number of proliferating cells from environmental atrazine exposure, supporting that there are no obvious defects in germ cell proliferation. Additionally, no changes were identified in sperm motility, highlighting that the decrease in *Ddx4* expression does not result in aberrations to sperm motility. Sperm are highly sensitive to hormonal disruptions, which is reported in many atrazine studies showing defects in sperm morphology, decreases in concentration and reduced sperm motility [10,11,17,19,20]. However, these studies again have used supra-environmental atrazine concentrations that are most likely toxic to sperm survival, whereas the atrazine concentration utilized in this study may not be high enough to cause a change to sperm development or motility. Equally, while expression of *Ddx4* may have changed due to an increase in gene transcription, this may not have resulted in a change in the protein level or enzyme activity. Hence, the changes reported here in *Ddx4* expression in the F1 generation are unlikely to result in biologically relevant effects on testis function.

The unique experimental design of the current study, looking at multigenerational effects only via maternal exposure, rather than each generation of males siring the next, to investigate the specific maternal contribution to the male reproductive phenotype likely accounts for some of the disparities in endpoint measures reported in previous atrazine exposure studies, beyond differences in dose and exposure timing. Thus, it is perhaps not surprising that this experimental design and the extensive endpoint measures analyzed resulted in only subtle changes induced by exposure to an environmental atrazine concentration. Hence, it would be anticipated that more prominent changes would be identified in a paternal lineage study, and certainly, a scenario where both parents are exposed, as would likely be the case in human and production animal exposures. Consequently, future studies should be undertaken using the same environmentally relevant atrazine concentration and extensive endpoint measures, but in these two experimental paradigms, as well as models where biological stressors (e.g., poor diet or advanced age) are incorporated to truly assess the possible consequences of chronic atrazine exposure. Finally, real world exposures likely involve multiple agents that may act synergistically with atrazine, and it is, therefore, necessary to investigate this reality before definitively concluding that exposure to low doses of atrazine does not negatively impact fertility.

## 5. Conclusions

The present novel maternal lineage study design and comprehensive endpoint measures demonstrated that chronic multigenerational exposure to an environmentally relevant atrazine concentration did not affect organ weights, testis morphology, germ cell proliferation or sperm motility, but did subtly alter early steroidogenesis gene expression in F1 and F2, as well as affect the expression of a germ cell-specific gene in F1 male mice. It is noted, however, that paternal lineage or combined parental atrazine exposure studies may result in more prominent effects on male offspring fertility. Hence, these studies should be undertaken in the future using the same environmentally relevant atrazine concentration and extensive endpoint measures to further elucidate the multigenerational effects of atrazine on male fertility.

## Figures and Tables

**Table 1:** The effect of atrazine-treated water (0.02 ng/mL) on body weight and organ weights (g) at 3 and 6 months of age in the F1 cohort. No significant differences were found between the control and ATZ treatment for all parameters. Data are mean ± SEM, with >7 L per treatment, per age cohort.

	Age = 3 Months		Age = 6 Months	
	Control (n = 19)	ATZ (n = 15)	Control (n = 17)	ATZ (n = 13)
Body weight (g)	27.85 ± 0.55	29.14 ± 0.55	35.17 ± 1.00	35.98 ± 1.35
Testes (g)	0.19 ± 0.01	0.19 ± 0.01	0.20 ± 0.01	0.20 ± 0.01
Seminal vesicle (g)	0.22 ± 0.01	0.25 ± 0.01	0.37 ± 0.02	0.40 ± 0.02
Liver (g)	1.18 ± 0.07	1.23 ± 0.07	1.48 ± 0.12	1.48 ± 0.13
Gonadal fat (g)	0.58 ± 0.05	0.68 ± 0.08	1.27 ± 0.14	1.43 ± 0.20

**Table 2:** The effect of atrazine-treated water (0.02 ng/mL) on body weight (g) and organ weights (g) at 3 and 6 months of age in the F2 cohort. No significant differences were found between the control and ATZ treatment for all parameters. Data are mean ± SEM, with > 7 L per treatment, per age cohort.

	Age = 3 Months		Age = 6 Months	
	Control (n = 12)	ATZ (n = 17)	Control (n = 12)	ATZ (n = 19)
Body weight (g)	29.09 ± 0.95	29.54 ± 0.62	34.74 ± 1.81	36.19 ± 0.71
Testes (g)	0.18 ± 0.01	0.19 ± 0.01	0.18 ± 0.01	0.20 ± 0.01
Seminal vesicle (g)	0.25 ± 0.01	0.27 ± 0.01	0.39 ± 0.02	0.42 ± 0.01
Liver (g)	1.08 ± 0.10	1.22 ± 0.06	1.37 ± 0.11	1.48 ± 0.06
Gonadal fat (g)	0.71 ± 0.12	0.76 ± 0.07	1.41 ± 0.19	1.45 ± 0.09

**Table 3:** The effect of atrazine on muscle, bone and fat properties at 3 and 6 months of age in the F1. Bone mineral density (BMD) values were multiplied by 10^4^ to reflect (mg/cm^2^). Bone mineral content (BMC) values were multiplied by 10^4^ to reflect milligrams (mg). No significant differences were found between the control and ATZ treatment for all parameters. Data are mean ± SEM, with >7 L per treatment, per age cohort.

	Age = 3 Months		Age = 6 Months	
	Control (n = 19)	ATZ (n = 15)	Control (n = 17)	ATZ (n = 13)
BMD (mg/cm^2^)	53.15 ± 0.60	54.33 ± 0.50	58.37 ± 0.86	58.09 ± 1.17
BMC (mg)	480.11 ± 10.94	493.87 ± 10.20	524.06 ± 9.94	520.46 ± 11.03
Fat (%)	17.06 ± 1.02	16.89 ± 1.34	25.06 ± 1.95	24.06 ± 2.23
Muscle (%)	82.94 ± 1.02	83.11 ± 1.34	74.94 ± 1.95	75.94 ± 2.23

**Table 4:** The effect of atrazine on muscle, bone and fat properties at 3 and 6 months of age in the F2. Bone mineral density (BMD) values were multiplied by 10^4^ to reflect (mg/cm^2^). Bone mineral content (BMC) values were multiplied by 10^4^ to reflect milligrams (mg). No significant differences were found between the control and ATZ treatment for all parameters. Data are mean ± SEM, with >7 L per treatment, per age cohort.

	Age = 3 Months		Age = 6 Months	
	Control (n = 14)	ATZ (n = 17)	Control (n = 10)	ATZ (n = 19)
BMD (mg/cm^2^)	55.90 ± 1.03	56.74 ± 0.63	58.84 ± 1.93	60.15 ± 0.92
BMC (mg)	475.43 ± 11.03	503.71 ± 9.31	507.88 ± 25.37	543.00 ± 7.55
Fat (%)	18.86 ± 1.45	18.34 ± 0.95	27.06 ± 2.65	26.01 ± 1.25
Muscle (%)	81.14 ± 1.45	81.67 ± 0.95	72.94 ± 2.65	73.99 ± 1.25

**Table 5:** The effect of atrazine on sperm motility at 6 months of age in the F1 and F2 measured using CASA. No significant differences were found between the control and ATZ treatment for all parameters. Data are mean ± SEM, with >3 L per treatment, per generation.

	F1		F2	
	Control (n = 7)	ATZ (n = 10)	Control (n = 10)	ATZ (n = 11)
Total motile sperm (%)	81.43 ± 3.34	85.60 ± 2.20	84.90 ± 2.09	83.18 ± 1.85
Progressive motile sperm (%)	50.43 ± 4.19	55.80 ± 2.80	54.70 ± 2.80	53.72 ± 2.49
Rapid velocity distribution (%)	54.86 ± 4.10	60.30 ± 2.70	59.30 ± 2.90	58.27 ± 2.53

**Table 6:** The effect of atrazine on seminiferous tubule measurements at 6 months of age in the F1. No significant differences were found between the control and ATZ treatment for all parameters. Data are mean ± SEM. >250 tubules were analyzed across >5 sections per animal (n = 4 control, n = 5 ATZ). There were >3 L per treatment.

	Control (n = 4)	ATZ (n = 5)
Epithelium height (µm)	65.85 ± 1.58	67.23 ± 1.15
Epithelium diameter (µm)	184.26 ± 4.82	189.30 ± 4.58
Tubule (%)	90.71 ± 0.74	91.34 ± 0.77
Interstitial space (%)	9.29 ± 0.74	8.66 ± 0.77

## Data Availability

The data presented in this study are currently available on request from the corresponding author.

## Appendix A

**Table A1:** Primer used in qRT-PCR experiments.

Gene	Accession Number	Primer Sequence	Product Length	Reference
*Tbp*	NM_013684.3	Forward—ACGGACAACTGCGTTGATTTT Reverse—ACTTAGCTGGGAAGCCCAAC	128	[19]
*β-actin*	NM_007393.5	Forward—CCACTGTCGAGTCGCGT Reverse—GTCATCCATGGCGAACTGGT	91	[19]
*Rpl19*	NM_001159483.1	Forward—AGGCTACAGAAGAGGCTTGC Reverse—GGCATTGGCGATTTCATTGGT	93	N/A
*Ddx4*	NM_010029.2	Forward—CCAAGATCAGGGGACACAGC Reverse—CTTTGGTAAGTGTCACCATTGC	90	N/A
*Cyp17a1*	NM_007809.3	Forward—CAATGACCGGACTCACCTCC Reverse—GGCAAACTCTCCAATGCTGG	124	N/A
*Cyp19a1*	NM_001348171.1	Forward—CGGGCTACGTGGATGTGTT Reverse—GAGCTTGCCAGGCGTTAAAG	135	N/A
*Gata6*	NM_010258.3	Forward—TTGCTCCGGTAACAGCAGTG Reverse—GTGGTCGCTTGTGTAGAAGGA	105	[59]
*Hsd17β3*	NM_008291.3	Forward—ACAAGATGACCAAGACCGCC Reverse—CCACAGGATTCAGCTCCGAT	76	N/A
*Hsd17β11*	NM_053262.3	Forward—CCTTGGGACGAACAGGAGTG Reverse—CCCGTGCATGAGATGTTCCA	134	[19]
*Hsd3β1*	NM_001304800.1	Forward—CCTCCTAAGGGTTACCCTATATCATACCAGCT Reverse—GTCTCCTTCCAACACTGTCACCTTGG	247	[60]
*Nr5a1*	NM_139051.3	Forward—CAGGAGCCCTGTCACTAAGC Reverse—ATGGTTAGAAGCCCCCTTTGG	184	[19]
*Srd5a1*	NM_175283.3	Forward—GTTTGCTCTGTTCACCCTGTG Reverse—TGGACAGCACACTAAAGCAGG	132	[19]
*Star*	NM_011485.5	Forward—TAAGGCAGCGCACTTGATCT Reverse—ACACAGCTTGAACGTAGCGA	119	N/A

Full gene names: TATA box binding protein (*Tbp*); Beta-actin (*β-actin*); Ribosomal protein L19 (*Rpl19*); DEAD-box helicase 4 (*Ddx4*); Cytochrome P450 family 17, subfamily A member 1 (*Cyp17a1*); Cytochrome P450 family 19, subfamily A member 1 (*Cyp19a1*); GATA binding protein 6 (*Gata6*); Hydroxysteroid 17-beta dehydrogenase 3 (*Hsd17β3*); Hydroxysteroid 17-beta dehydrogenase 11 (*Hsd17β11)*; Hydroxy-delta-5-steroid dehydrogenase, 3 beta-and steroid delta-isomerase 1 (*Hsd3β1*); Nuclear receptor subfamily 5 group A member 1 (*SF-1; Nr5a1*); Steroid 5 alpha-reductase 1 (*Srd5a1*); Steroidogenic acute regulatory protein (*Star*); and N/A, not applicable.
